# Conservative Management of Acalculous Cholecystitis in a Seven-year-old Child

**DOI:** 10.7759/cureus.2092

**Published:** 2018-01-20

**Authors:** Jessica Y Ng, Jennie Gu

**Affiliations:** 1 Surgery, Gold Coast University Hospital

**Keywords:** acalculous cholecystitis, paediatric, surgery

## Abstract

Acute acalculous cholecystitis is an uncommon disease in children and is usually associated with trauma, burns, and infections. Whereas acute acalculous cholecystitis is only seen in 10% of cholecystitis in adults, it is uncommon in the paediatric population.

A seven-year-old male presented to the emergency department of a regional hospital with a 36-hour history of right-upper-quadrant abdominal pain. He had associated symptoms of anorexia, nausea, and vomiting. He was septic with raised white cell count and inflammatory markers. Diffuse gallbladder wall thickening without intraluminal sludge or calculi was seen on abdominal ultrasound. He was found to have a concurrent right-upper lobe pneumonia on further investigation. The patient was treated with antibiotics and responded well to supportive and conservative management with close radiological monitoring.

Acute acalculous cholecystitis is associated with a high mortality rate (30%) and significant complications such as gangrene, empyema, and perforation in 40% of adult cases. Acute surgical management has been traditionally advocated, however, surgery is not without risks; studies have suggested that non-operative intervention may be appropriate for selected critically ill children with an underlying cause.

Herein, we discuss the safe and effective conservative treatment of acute acalculous cholecystitis in lieu of operative management and highlight the importance of recognising this disease in paediatric patients with acute abdominal pain and coexisting infection.

## Introduction

Calculous cholecystitis is a rare disease in children, with 1.3 paediatric cases for every 1000 adult cases [1]. Acalculous cholecystitis (AC) is even more uncommon, comprising only 50% of these paediatric cholecystitis cases [2]. This condition is associated with bacterial (Salmonella, Brucella, Campylobacter, Leptospira), fungal (Candida), viral (Hepatitis A and B, Epstein–Barr virus, Cytomegalovirus) and parasitic infections (Giardiasis, Malaria, Echinococcus, Ascaris Lumbricoides) [3-7]. It is related to trauma, burns, postoperative patients, and total parenteral nutrition [3,8-10]. Cases have been described in autoimmune conditions such as Kawasaki’s disease, Periarteritis nodosa and Systemic Lupus Erythematosus [10]. It can also occur in otherwise healthy children.

## Case presentation

A seven-year-old, Caucasian boy was bought into the Emergency Department with 36-hour history of right-sided abdominal pain associated with nausea, anorexia, and vomiting. He reported no coryzal symptoms or changes in bowel motions and medical history was unremarkable. He had no perinatal or developmental issues and all immunisations were up to date.

On examination, the child looked well, was warm and well perfused. He was not tachypnoeic and was saturating at 98% on room air. He was febrile to 39 degrees on arrival, with a heart rate of 130. Chest sounds were equal and bilaterally vesicular and abdomen was soft but distended. He had generalised tenderness, worst in the right upper and lower quadrants. Bowel sounds were present and external genitalia were unremarkable.

**Figure 1:**
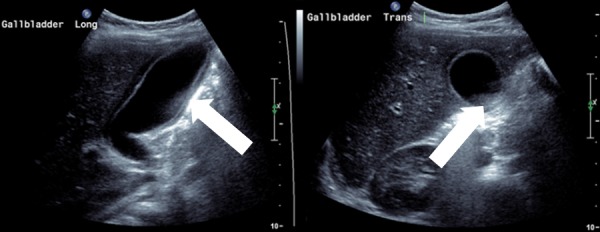
Ultrasound gallbladder – diffuse gallbladder wall thickening with pericholecystic collection. No intraluminal sludge or calculi seen.

**Figure 2:**
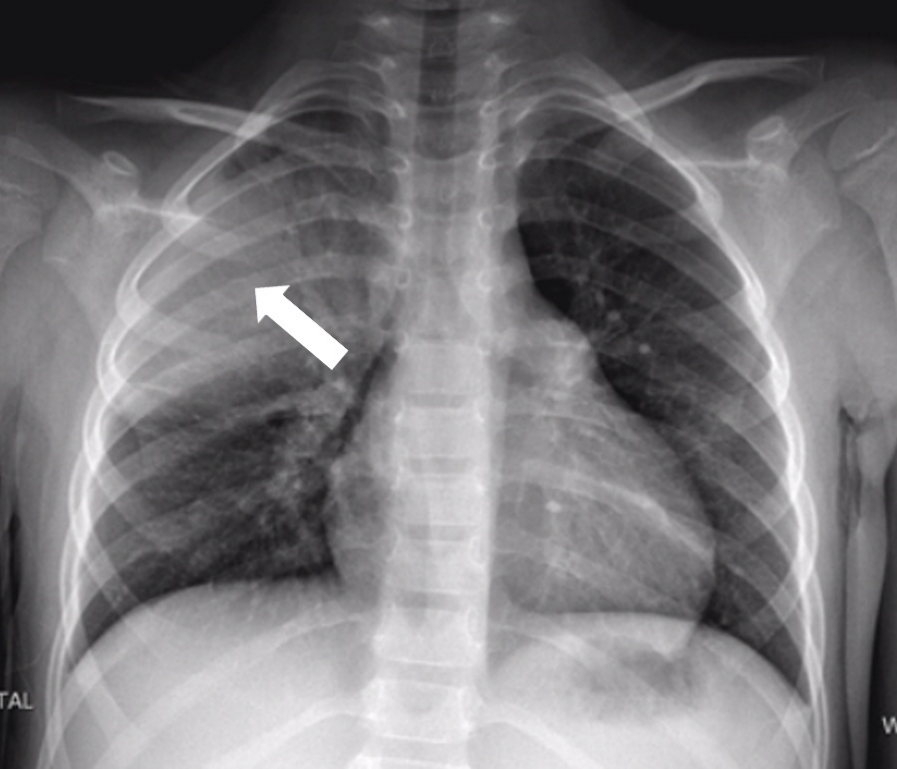
Chest X-ray – large focal area of consolidation seen in the right upper mid zone, consistent with right-upper lobe pneumonia.

A urine microscopy was unremarkable. Initial blood showed a haemoglobin of 109 g/L, white cell count of 15.5 x 10^9/L, platelets of 203 x 10^9/L and C reactive protein of 301 mg/L. His albumin was 25 g/L (range 32-47), total bilirubin was 20 umol/L (range <20) and conjugated bilirubin was 6 umol/L (range <4). His electrolytes, other liver function tests (LFTs) and lipase were unremarkable. An abdominal ultrasound showed a mildly enlarged, heterogenous liver with no focal abnormality. Diffuse gallbladder wall thickening up to 4.6 mm with pericholecystic collection was identified - no intraluminal sludge or calculi seen (Figure 1). The common bile duct measured 2 mm. A chest X-ray revealed a large focal area of consolidation seen in the right upper mid zone, consistent with right-upper lobe pneumonia. Cardiac and mediastinal silhouette were within normal limits (Figure 2).

The patient was initiated on ceftriaxone and metronidazole. Bilirubin normalised and LFTs remained normal during admission. Blood cultures were negative. Symptoms improved over the next few days and a repeat ultrasound showed resolution of cholecystitis. After five-day of IV antibiotics, he was discharged home with a five-day course of amoxycillin and clavulanic acid.

**Figure 3:**
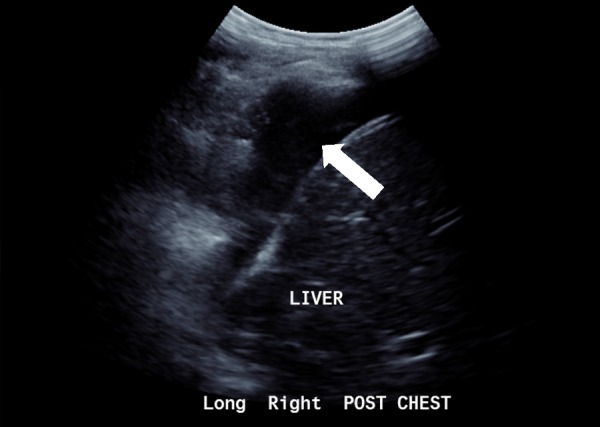
Ultrasound chest – complex septated right-sided effusion with associated low level internal echoes.

He was readmitted two days later with worsening respiratory symptoms and ongoing fevers due to worsening pneumonia and development of a right-sided multi-loculated pleural effusion (Figure 3). Video-assisted thoracoscopic surgery and breakdown of locules were performed and an intercostal catheter was inserted. He was treated empirically with IV lincomycin and cefotaxime until sensitivities of pleural cultures were revealed a Staphylococcus warneri which was sensitive to flucloxacillin. His blood anti-streptolysin O titre was 1150 U/mL (range <200). Serial abdominal ultrasound scan revealed no recurrence of his cholecystitis. He was discharged on oral amoxicillin after a four-day admission and was reviewed in outpatient department three weeks later with complete resolution of all symptoms.

## Discussion

Acute acalculous cholecystitis (AAC) is associated with a high mortality (30%), with complications of gangrene, empyema, and perforation occurring in 40% of adult cases [2,3]. Hence acute surgical management has been traditionally advocated.

Diagnosis is complicated by these other underlying diseases, and hence is often delayed. The exact cause remains unclear. Ultrasonography is the diagnostic imaging procedure of choice, as it is portable, noninvasive, relatively inexpensive, and bears no radiation risk to the paediatric patient [8].

Therapeutic options advocated for AAC include cholecystectomy or cholecystostomy [3]; traditionally cholecystectomy is the procedure of choice. Tsakayannis et al., in their series of 12 patients, treated the majority of patients with cholecystectomy [9]. They suggested non-operative intervention may be appropriate for selected critically ill children with an underlying cause, i.e., infection. In a similar study of 12 patients by Imamoglu et al., they advocated initial non-operative treatment with serial ultrasound scan to determine the most favourable time for operative intervention [8].

Other studies showed management with appropriate antibiotics and supportive therapy to be adequate. Thambidorai et al. studied the outcomes of nonsurgical management of AAC in a series of 14 cases associated with enteric fever [7]. All patients recovered uneventfully without the need for surgery [7]. Gora-Gebka et al. described two cases of AAC secondary to viral illness (Epstein–Barr virus and cytomegalovirus) which were successfully treated conservatively [5].

## Conclusions

In conclusion, AAC needs to be considered as a differential in all children who present with acute abdominal pain, particularly in the setting of other infective illness, trauma or surgery. Conservative, supportive management and treatment of the underlying condition may be safe and effective in lieu of operative management.
